# Correction: Near point-of-care HIV viral load testing: Cascade after high viral load in suburban Yangon, Myanmar

**DOI:** 10.1371/journal.pone.0331295

**Published:** 2025-08-29

**Authors:** Ni Ni Tun, Frank Smithuis, Nyan Lynn Tun, Myo Min, Myo Ma Ma Hlaing, Josefien van Olmen, Lutgarde Lynen, Tinne Gils

An additional affiliation is missing for the first author. Ni Ni Tun is also affiliated with the Department of Family Medicine and Population Health, University of Antwerp, Antwerp, Belgium.
