# Supplementary material for: Near point-of-care HIV viral load testing: Cascade after high viral load in suburban Yangon, Myanmar
Source: PLoS One. 2023 Apr 13;18(4):e0272673. doi: 10.1371/journal.pone.0272673 (PMC10101411; doi:10.1371/journal.pone.0272673)
Supplement: Attachment — Submitted filename: Response to Reviewers.docx [file pone.0272673.s003.docx]

Dear editor, reviewers,

We thank you for your time to review our manuscript for the second time and provide comments for improvement. Please find with this “Response to reviewers” the following documents for your review:

- The marked revised manuscript: 'Revised Manuscript with Track Changes'.
- The unmarked version: 'Manuscript'.

In this letter, we have provided our feedback to your comments and suggestions in blue.

Reviewer 1

Adding “Group 1” type labels for the three groups was suggested and these labels are introduced in the methods on page 6. However, it would help Figure 2 to add the group names in the legends within the graphs (such as Group 1 No VL).

The new figure 2, with labels, is submitted with this rebuttal.

The overall legend for Figure 2 needs more explanation of the difference between A and C, and the difference between B and D. Here is a suggested addition to this figure legend: Panels A and B include all three groups while Panels C and D only compare Groups 2 and 3.

We have replaced the legend of figure 2 with the following legend:

**Figure 2. Comparing outcomes on ART among PLHIV with different access to VL testing**

Panel A, C present two-year attrition (dead, lost-to-follow-up) and panel B, D two-year mortality between PLHIV on ART who, when becoming eligible, had no access to VL testing (Group 1: No VL), access to laboratory-based VL testing (Group 2: Laboratory-based VL) or access to near POC VL testing (Group 3: Near POC VL). Panels A and B include all three groups while Panels C and D only compare Groups 2 and 3.

ART = antiretroviral therapy, PLHIV = people living with HIV/AIDS, POC = point of care, VL = viral load

Table 3: add the explanation for “part of key population” to Table 3 that was used in Table 1 so the reader does not need to remember.
This was added in the legend of table 3.

Reviewer #2:

In this manuscript, Tun et al present an updated report describing the implementation of POC VL testing in Burma. Thank you so very much for an extremely thoughtful revision to the original comments.

Thank you.

Seeing these data, I only have one comment:
In new Table 2, there are two main conclusions one could draw. The first is the very clear finding that more people received VL testing when POC testing was available. The second, however, is that people who had VL testing done and who had a VL>1000 copies - they were more likely to have documented suppression if their testing was done by the lab c/w POC testing - this is a bit counter-intuitive.

If I'm reading the data correctly there were 49-23= 26 (49%) people out of the 53 with >1000 copies in 2013-2017 who had a documented suppressed VL after EAC and only 125-84 = 41 (28%) out of 149 after 2017 -p = 0.004.

Are there possible explanations for this?

Thank you.

During the laboratory-based VL testing period, there was limited testing capacity. Hence, targeted VL testing prioritized PLHIV with demonstrated adherence problems, who could have had more benefit from the EAC sessions. In the near-POC era, back-log cases who had never had a VL were tested, who might have been failing for a long time. That could explain less re-suppression after EAC, due to true treatment failure. That could explain more patients receiving a follow-up VL result of ≤1000 copies/ mL after EAC in the laboratory-based testing era than in the near-POC era.

We have added a line in the results (lines 258-60 in Manuscript) stating:

“Among PLHIV with a first VL>1000 copies/mL between 2013 and 2017, and those after 2017, respectively, 49% (26/53) and 28% (41/125) had a suppressed follow-up VL (p= 0.004).”

In the discussion, we have added (lines 334-40 in Manuscript):

“Among PLHIV with a first high VL after near POC implementation, less had a suppressed follow-up VL documented compared to those who had a first high VL before. This could be explained by the fact that due to the limited laboratory-based testing capacity, targeted VL testing prioritized PLHIV with demonstrated adherence problems. This population could thus have benefitted more from the EAC sessions. In the near-POC era, all back-log cases who had never had a VL were tested, among whom more might have experienced true treatment failure”.
